# Decoding sugarcane smut: the role of effector SsEF83 in fungal virulence and plant interaction

**DOI:** 10.3389/fmicb.2025.1586720

**Published:** 2025-08-18

**Authors:** Xuecheng Huang, Zongling Liu, Yizu Su, Jinfeng Qiu, Haoming Wu, Zhenhua Ming, Ru Li

**Affiliations:** ^1^State Key Laboratory for Conservation and Utilization of Subtropical Agro-Bioresources, College of Life Science and Technology, Guangxi University, Nanning, China; ^2^College of Basic Medical Sciences, Youjiang Medical University for Nationalities, Baise, China; ^3^College of Agriculture, Guangxi Key Laboratory of Sugarcane Biology, Guangxi University, Nanning, China

**Keywords:** *Sporisorium scitamineum*, effector, cell death, virulence, sugarcane smut, SsEF83

## Abstract

Sugarcane smut, caused by the fungus *Sporisorium scitamineum*, severely affects global sugarcane production. Effector proteins are crucial for fungal invasion and virulence in plants. Nevertheless, few studies have identified effector proteins in *S. scitamineum* and demonstrated their functions in smut progression. This study aimed to characterize SsEF83 (SPSC_06083), a cysteine-rich core effector of *S. scitamineum*. Our qRT-PCR results revealed that the expression of *SsEF83* was significantly upregulated at 24, 72, and 168 h post-infection in sugarcane. Further confirmation of the secretion function of the SsEF83 signal peptide was provided by the yeast secretion system. SsEF83 was found to induce cell death in *Nicotiana benthamiana* leaves. Subcellular localization analysis indicated that SsEF83 protein was localized in the cytoplasm. Notably, the deletion of *SsEF83* did not alter fungal morphology, growth, and mating/filamentous, but it significantly attenuated the virulence of *S. scitamineum*. Furthermore, yeast two-hybrid screening was employed to identify sugarcane proteins interacting with SsEF83. Five sugarcane proteins were found to interact with SsEF83, and these interactions were confirmed by Bimolecular Fluorescence Complementation (BiFC). Through AlphaFold 3 analysis of the interactions between SsEF83 and target proteins, significant binding interactions were detected between SsEF83 and target-2 or target-4. Moreover, our previous RNA-seq data suggested a potential association between the two target proteins and sugarcane's susceptibility or resistance to smut. Collectively, our findings demonstrated that the effector SsEF83 plays a crucial role in the virulence of *S. scitamineum*, providing new insights into the interaction between smut fungi and sugarcane.

## Introduction

Sugarcane is the world's primary sugar crop, accounting for over 80% of global sugar production. It is widely cultivated in more than 120 countries, primarily in tropical and subtropical regions. The sugarcane industry, however, faces significant yield losses due to sugarcane smut disease, caused by *Sporisorium scitamineum* (Rajput et al., 2021). This pathogen is a biotrophic fungus that infects sugarcane through the stem buds. At the beginning of the infection cycle, fungal teliospores germinate to produce haploid basidiospores. Two opposite mating types of basidiospores fuse to form the infective dikaryotic mycelium. Once *S. scitamineum* colonizes sugarcane, its hyphae grow within the shoot meristem, ultimately leading to the formation of a characteristic black whip structure composed of mature teliospores and plant debris (Bhuiyan et al., 2021).

It is well-known that plants activate defense responses upon pathogen challenge, which are broadly categorized into two types: pathogen-associated molecular pattern-triggered immunity (PTI) and effector-triggered immunity (ETI; Stergiopoulos and De Wit, 2009). Pathogens secrete effector proteins into host cells to suppress PTI and disturb normal physiological activities, thereby promoting infection (De Wit et al., 2009; Stergiopoulos and De Wit, 2009; Lo Presti et al., 2015). In the process of ETI, plants typically recognize effectors directly or indirectly through nucleotide-binding domain leucine-rich repeat (NLR) receptors. This recognition triggers a robust immune response, often involving programmed cell death (PCD). This response manifests as a hypersensitive response (HR), which effectively limits pathogen invasion and spread (Boller and He, 2009; Katagiri and Tsuda, 2010; Van Der Burgh and Joosten, 2019). For example, the effector Avr1 of *Phytophthora infestans* interacts with host protein Sec5 to inhibit INF1-induced PCD (Du et al., 2015). The phylotype II strain of *Ralstonia solanacearum* overcomes the resistance of the tomato cultivar Hawaii 7996 by utilizing the type III effector protein RipV2, an E3 ubiquitin ligase, which targets key immune components in tomato, including SlNRG1, EDS1, and SAG101b. Through this process, RipV2 inhibits Toll/interleukin-1 receptor/nucleotide-binding leucine-rich repeat (TNL)-mediated cell death and immune responses, thereby breaking the resistance of the disease-resistant tomato variety Hawaii 7996 (Qi et al., 2024). The oomycete effector HaRxL10 directly hijacks the Arabidopsis central clock component CCA1 HIKING EXPEDITION (CHE) to repress plant immunity and manipulate physiological processes (Fu et al., 2025).

Many smut effectors exhibit enzymatic activities, such as isomerase, peroxidase, and protease functions (Doehlemann et al., 2009, 2011; Djamei et al., 2011; Hemetsberger et al., 2012; Mueller et al., 2013; Ma et al., 2018; Schweizer et al., 2018). However, many smut-secreted effectors lack identifiable functional domains (Schuster et al., 2018). As a phytopathogenic fungus with characteristics of systematic infection, *S. scitamineum* secretes effectors to facilitate host colonization (Teixeira-Silva et al., 2020). In 2015, (Taniguti et al. 2015) published the complete genome sequence of *S. scitamineum*, revealing 26 chromosomes, 6,677 genes, and 527 predicted effector proteins. Despite this, the functional characterization of *S. scitamineum* effectors remains largely limited. For instance, (Teixeira-Silva et al. 2020) cloned 10 effector protein genes and investigated their expression levels, subcellular localization, and interacting proteins in smut-resistant sugarcane varieties. (Lu et al. 2021b) identified the multifunctional effector SsPEP1, which is essential for both sexual mating and pathogenicity in *S. scitamineum*. (Maia et al. 2022) used the type III secretion system of *Pseudomonas fluoresces* to deliver six candidate effector proteins into tobacco cells, demonstrating that effector proteins g1052 and g5159 simultaneously suppress the PTI and ETI immune responses of plants. (Ling et al. 2022) revealed that the effector protein Pele1 is secreted into the apoplast space of plants during the infection of sugarcane by *S. scitamineum*. Pele1 mimics the host plant Pep1 through terminal peptides to compete for binding to the PEPR1 receptor, thereby inhibiting immune responses triggered by damage-associated molecular patterns mediated by the peptide-kinase complex (PEP1-PEPR1). This interaction reduces host resistance and promotes pathogen infection and proliferation. Despite these advances, the identification of effector proteins in *S. scitamineum* and their interaction mechanisms with the host are still not fully understood compared to other pathogenic fungi.

Recently, dual RNA-seq has emerged as a powerful tool for dissecting host-pathogen interactions, as demonstrated in studies of *Magnaporthe oryzae* and rice (Yan et al., 2023), *Mycosarcoma maydis* and maize (Lanver et al., 2018), *Leptosphaeria maculans* and oilseed rape (Gay et al., 2021), *Phytophthora palmivora* and tobacco (Evangelisti et al., 2017), and *Botrytis cinerea* and *Arabidopsis thaliana* (Wei et al., 2024). In our previous work, we employed dual RNA-seq combined with Weighted Gene Co-expression Network Analysis (WGCNA) to investigate the early stages of smut infection in sugarcane. Through this analysis, we identified several core smut effector proteins that are potentially critical to the infection process of *S. scitamineum* (Liu et al., 2022). In this study, we characterized SsEF83 (SPSC_06083), a cysteine-rich core effector of *S. scitamineum*. Using a transient expression system in *Nicotiana benthamiana* and generating *SsEF83* deletion mutants in *S. scitamineum*, we explored its biological functions. Additionally, we screened putative host target proteins of SsEF83 using a yeast two-hybrid assay. Furthermore, the association between the SsEF83 and target proteins was confirmed by bimolecular fluorescence complementation assay. In conclusion, our findings provide new insights into the functional roles of SsEF83 in smut fungi, advancing our understanding of effector biology in *S. scitamineum*.

## Material and methods

### Plant materials and test strains

For this experiment, the sugarcane smut-susceptible variety ROC22 and smut-resistant variety ZZ1 were selected and cultivated under standard management practices in the open field at the Guangxi University sugarcane germplasm nursery. The *N. benthamiana* used in this study was procured from our laboratory and cultivated under controlled environmental conditions within a designated artificial climate chamber. The chamber maintained a temperature of 25°C and a relative humidity of 60%, with a photoperiod (16 h of light followed by 8 h of darkness).

The *S. scitamineum* strains JG36 (mating type 1, Mat-1) and JG35 (mating type 2, Mat-2) were isolated and preserved in our laboratory. These fungal strains were grewon solid YEPS medium (1% yeast extract, 2% peptone, 2% sucrose, 1.5% agar) or in liquid YEPS medium at 28°C. Mating and filamentation assays, along with growth curve analyses of *S. scitamineum*, were performed as previously described (Deng et al., 2018). The *Escherichia coli* strain DH5α (AngYuBio, Shanghai, China) was cultured on Luria agar (LA) plates or in Luria broth (LB) at 37°C. The *Agrobacterium tumefaciens* strain GV3101 (AngYuBio, Shanghai, China) was grown in YEP medium (1% beef extract, 1% yeast extract, 0.5% NaCl, pH 7.0). The yeast strain YTK12, using for signal peptide activity validation, was preserved in our laboratory and grew on YPDA medium (1% yeast extract 10 g, 2% peptone 20 g, 2% D-glucose 20 g, 0.2% adenine 15 mL, 1.5% agar) at 30°C.

### Transient expression in *N. benthamiana* and fluorescence imaging

The *SsEF83* gene was amplified from *S. scitamineum* JG35 cDNA with primers containing *Bam* HI and *Sac* I (Supplementary Table S1). The fragment was ligated into expression vector pCAMBIA3300-CaMV 35S. The competent cells of *A. tumefaciens* strain GV3101 (AngYuBio, Shanghai, China) were transformed with recombinant vectors pCAMBIA3300*-SsEF83* following the manufacturer's instructions. Colonies with recombinant constructs were grown overnight in YEP medium containing 50 μg/mL kanamycin and 20 μg/mL rifampicin. The cells were resuspended twice in an infiltration buffer composed of 10 mM MgCl_2_, 10 mM MES, and 150 μM acetosyringone, and subsequently infiltrated into 4–5-week-old *N. benthamiana* leaves at OD_600_ = 0.6. Leaf apoptosis was observed after 5 days of infiltration.

For the subcellular localization assay, the full-length CDS of SsEF83 was cloned and fused with GFP in the pCAMBIA3300*-SsEF83* vector. This construct was co-infiltrated with a Golgi-Marker into *N. benthamiana* leaves for transient expression. Two days after infiltration, the subcellular localization of the effectors was visualized in the plant cell using laser confocal microscopy (model TCS-SP8 MP, Leica, Germany) using a 488 nm excitation laser for GFP and a 558 nm laser for RFP.

### Confirmation of the secretion function of signal peptide

The sequence of SsEF3's signal peptide predicted by SignalP (https://services.healthtech.dtu.dk/services/SignalP-5.0/) was amplified by PCR and cloned into the *EcoR* I and *Xho* I sites of a pSUC2 vector to construct the plasmid pSUC2-*SsEF83*sp and then transformed into yeast strain YTK12 through the lithium acetate method. After 2 days of growth on the CMD-W medium [0.67% YNB (yeast nitrogen base without amino acids), 0.075%/-Trp DO supplement, 2% sucrose, 0.1% glucose, 1.5% agar], yeast cells carrying fused pSUC2 vectors were examined on the YPRAA medium (1% yeast extract, 2% peptone, 2% raffinose, 1.5% agar). Quantification of the activities of sucrose invertase was performed using TTC (2,3,5-Triphenyltetrazolium Chloride) as substrate according to the previous report (Oh et al., 2009). Specifically, yeast colonies successfully grew on CMD-W medium plates were re-cultured in CMD-W liquid medium at 30°C for 36 h. 5 mL of yeast culture was centrifuged at 12,000 *g* for 1 min and washed twice with ddH_2_O. The yeast cells were resuspended in 1.5 mL of ddH_2_O, 500 μL of 10 mM acetic acid-sodium acetate buffer (pH = 4.7) and 1 mL of 10 % sucrose solution. The suspension was incubated at 37°C for 10 min and then centrifuged at 12,000 *g* for 1 min. 400 μL of the supernatant was taken and co-incubated with 3.6 mL of 0.1% TTC at room temperature for 5 min or longer until the red color could be observed.

### DNA and RNA extraction, quantitative real-time quantitative PCR

*S. scitamineum* strains were cultured on solid YEPS plates at 28°C for 3 days. Genomic DNA was extracted using the MiniBEST Plant Genomic DNA Extraction Kit (TaKaRa, Beijing, China). Total RNA was extracted from fungal cells using TransZol reagent (TransGen Biotech, Beijing, China). Primers for quantitative real-time PCR (qRT-PCR) were designed using Primer Blast (https://www.ncbi.nlm.nih.gov/tools/primer-blast/index.cgi?LINK_LOC=BlastHome; Supplementary Table S1). Reverse transcription and qPCR were conducted with BeyoRT III cDNA synthesis premix (5 × ; with gDNA EZeraser; Beyotime, D7185M) and ChamQ Universal SYBR qPCR Master Mix (Vazyme, Q711) following the manufacturer's instructions. Housekeeping genes of *S. scitamineum*, encoding inosine 5′-monophosphate dehydrogenase (*S10*) and SEC65-signal recognition particle subunit (*S11*) or *Actin* were used to normalize expression (Huang et al., 2018). Three biological replicates and three technical replicates for each sample. Relative expression ratios were obtained using the 2^−ΔΔ*Ct*^ method (Livak and Schmittgen, 2001).

### Bioinformatics analysis

The SsEF83 protein sequence was used as query sequence to perform BLASTp in the NCBI database. The paralogs of the SsEF83 were predicted by blasting against the *S. scitamineum* genome using the protein sequence of experimentally-verified effector. Sequence alignment and phylogenetic tree construction were performed using the ClustalW and maximum likelihood methods in MEGA7 (Kumar et al., 2016), respectively. Conserved motifs of sequences used for phylogenetic tree construction were predicted using the TBtools v1.09 software (Chen et al., 2020). Phylogenetic trees and Circos plots were visualized using the TBtools v1.09 software. Cysteine content was analyzed using DiANNA v1.1 (http://clavius.bc.edu/~clotelab/DiANNA/). The motifs of SsEF83 effector protein were predicted using MEME software [MEME—MEME Suite (meme-suite.org)], followed by further analysis of conserved sites within protein domains. The interaction prediction of target proteins was conducted using AlphaFold 3 (https://alphafoldserver.com/), and the prediction results were visualized using PyMol software.

### Generation of *SsEF83* knockout mutants in *S. scitamineum*

The *SsEF83* knockout mutants were constructed through homologous recombination (Cai et al., 2021). The genomic DNA of JG35 was used as a template to amplify the left and right homologous arms (LB and RB) of *SsEF83* using primer pairs (Supplementary Table S1). Similarly, the left and right homologous arms of the hygromycin phosphotransferase gene (HPT-LB, HPT-RB) were amplified from the pDAN plasmid using specific primers (Supplementary Table S1). The left homologous arm was fused to HPT-LB, and the right homologous arm to HPT-RB through fusion PCR. Subsequently, the resulting homologous fragments were transformed into the JG35 and JG36 protoplast, respectively. The knockout mutants were screened by hygromycin resistance, and the knockout results of the gene deletion mutants were determined by PCR detection using the relevant primers. The primers used in this experiment are shown in Supplementary Table S1.

### Generation of *SsEF83* complement transformants

To generate complementary transformants of Δ*SsEF83*, we inserted *SsEF83* and its promoter into the vector pEASY (*Pme* I). The recombinant plasmid pEASY*-SsEF83* was obtained, and the left-arm fragment (Com-LB-SsEF83, containing the promoter and SsEF83 gene DNA sequence) and right-arm fragment (Com-RB-SsEF83) for complementation were amplified using primer pairs listed in Supplementary Table S1. Subsequently, the amplified Com-LB-SsEF83 and Com-RB-SsEF83 fragments were co-transformed into Δ*SsEF83* protoplasts via polyethylene glycol-mediated protoplast transformation. All these transformants were screened for Zeocin resistance and further confirmed by PCR.

### Pathogenicity assay

Pathogenicity assays were performed using the root dipping method as reported in earlier studies (Lu et al., 2021a). In brief, sugarcane ROC22 plantlets derived from tissue culture were immersed in *S. scitamineum* basidiospore cell suspension (1 × 10^6^ cells/mL) and incubated at 28°C for 3 days. Subsequently, the seedlings were transplanted into pots filled with nursery substrate and maintained in a growth chamber at 26–28°C under a 16-h light/8-h dark cycle with 80–85% relative humidity. Infection rates were determined by calculating the ratio of the number of whip seedlings to the total number of inoculated seedlings. Sugarcane tissue samples were stained with 0.4% trypan blue (Lu et al., 2021a) and visualized under an Olympus BX51 microscope.

### Yeast two-hybrid assay

The cDNA fragment encoding the *SsEF83* gene without signal peptide was inserted into the pGBKT7 (*BamH* I and *EcoR* I) plasmid to serve as a bait vector. The recombinant constructs of pGBKT7-*SsEF83* (SP loss) were transformed into competent Y2H Gold yeast cells following the manufacturer's instructions. For the autoactivation tests of SsEF83 bait constructs, the recombinant plasmid pGBKT7-*SsEF83* and the pGADT7 empty plasmid were co-transformed into Y2HGold yeast competent cells. Then the yeast cells were grown on SD/-Trp/-Leu medium and SD/-Trp/-Leu/-Ade/-His medium at 30°C for 2–3 days.

The prey libraries were constructed using the cDNA from sugarcane infected with smut fungus in Y187 yeast (Oebiotech, Shanghai, China). Following the yeast protocol manual provided by Clontech, the bait Y2H and Y187 yeasts were mated on YPDA medium for 24 h and then transferred to SD/-Trp/-Leu/-His medium. The positive interactions were analyzed by selecting cells using media SD/-Trp/-Leu/-Ade/-His+X-α-Gal. To validate the interaction between SsEF83 and its targets, the recombinant pGBKT7-*SsEF83* and pGADT7-Prey vectors were co-transformed into yeast strains and cultured on SD/-Trp/-Leu/-Ade/-His+X-α-Gal medium, respectively. The co-transformation of pGADT7-T7 and pGBKT7-p53 served as the positive control, while the co-transformation of pGBKT7-*SsEF83* and empty pGADT7 vector served as the negative control. The primers are listed in Supplementary Table S1.

### Bimolecular fluorescence complementation

For the bimolecular fluorescence complementation assay, *SsEF83* was subcloned into the pCAMBIA1301-cYFP vector (*Sma* I). The interacting protein sequences were amplified from sugarcane ROC22 cDNA using primer pairs (Supplementary Table S1) and subsequently ligated into the pCAMBIA1301-nYFP vector (*Sma* I). The recombinant constructs were then transformed into *A. tumefaciens* strain GV3101 competent cells, respectively. The successfully transformed *A. tumefaciens* cells were incubated overnight at 28°C. Bacteria were then collected by centrifugation, washed twice with infiltration buffer and finally resuspended in infiltration buffer to the concentration of OD_600_ = 1. Four-week-old *N. benthamiana* leaves were injected with different combinations of bacterial suspension for transient protein expression. Two days post-infiltration, fluorescence in the *N. benthamiana* leaves was detected using bioluminescence imaging (IVIS Lumina LT, PerkinElmer). Co-infiltration of 35S::nYFP-T7 and 35S::cYFP-p53 served as the positive control, while co-infiltration of 35S::nYFP and 35S::cYFP served as the negative control. Each experiment was conducted in triplicate.

### Statistical analysis

Statistical significance was determined by analysis of variance (ANOVA) using SPSS software (version 26), with significance levels denoted as follows: ^*^p < 0.05, ^**^p < 0.01, ^***^p < 0.001, and ^****^p < 0.0001; results without annotation were considered statistically non-significant.

## Results

### Identification of the core effector *SsEF83* in *S. scitamineum*

**Figure 1:**
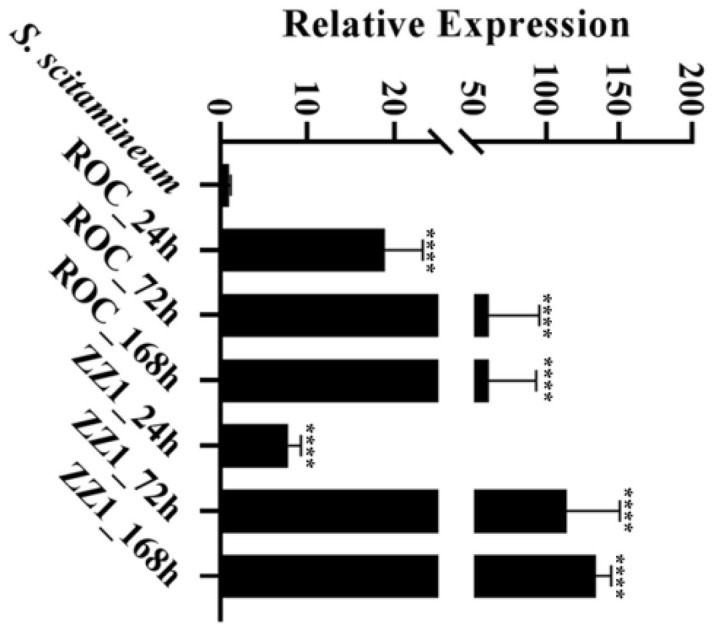
The expression of SsEF83 was highly upregulated in planta. The smut-resistant variety ZZ1 and smut-susceptible variety ROC22, both inoculated with *S. scitamineum* wild-type strain, were harvested at 24, 72, and 168 h after infection. Total RNA was used for qRT-PCR. Haploid strains of JG35 and JG36 cultured *in vitro* (labeled as *S. scitamineum*) were used as control. Three biological replicates and three technical replicates were used for each sample. The asterisk indicates a statistically significant difference from *S. scitamineum* (^****^p < 0.0001; ANOVA with Tukey's multiple comparisons test).

Based on our previous dual RNA-seq combined with WGCNA results, we identified several core effector proteins potentially involved in regulating the infection process of *S. scitamineum* (Liu et al., 2022). Among these, a cysteine-rich core effector in *S. scitamineum, SsEF83* (SPSC_06083), drew our particular attention. The *SsEF83* gene was significantly induced according to RNA-seq data when sugarcane was infected by *S. scitamineum*. To further verify the expression pattern of *SsEF83* in different stages of infection, the RNA of sugarcane ROC22 and ZZ1 infected with *S. scitamineum* strain for 24–168 h was extracted, respectively. qRT-PCR analysis revealed that the expression of *SsEF83* was significantly upregulated at 24, 72, and 168 h after infection, and the expression level was the highest at 168 h in ZZ1 (Figure 1). These findings showed that *SsEF83* may played a crucial role in the interaction between *S. scitamineum* and host sugarcane.

### Characterization of the core effector *SsEF83*

The SsEF83 cDNA contains an open reading frame with 552 bp in length, encoding a putative protein with 183 amino acid residues. The *SsEF83* gene is located on scaffold 44 of *S. scitamineum*. The secretion of SsEF83 protein was predicted by SignalP, with signal peptide containing 29 amino acids in length (Supplementary Figure S1). Besides the secretion signal, no known protein domains were found within SsEF83.

**Figure 2:**
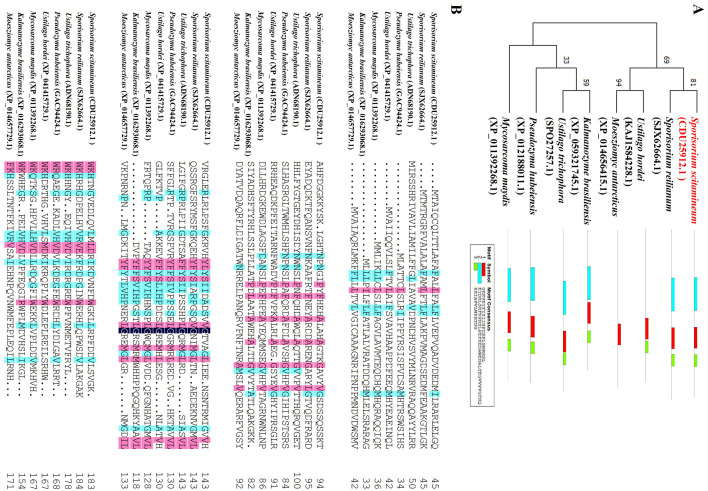
Phylogenetic analysis and amino acid sequences arrangement with SsEF83 protein and its orthologs. **(A)** Phylogenetic tree and domain analysis of SsEF83 homologs in different fungi constructed using MEGA7. The phylogenetic tree based on full-length sequence alignment of SsEF83 homologs from different fungi was generated using the maximum likelihood method in MEGA7 software with 1000 bootstrap replicates. The scale represents the number of substitutions per site. The numbers next to the species' names correspond to the protein accession numbers in NCBI. Domain prediction of SsEF83 was performed using MEME. **(B)** Multiple sequence alignment was generated using DNAMAN with the following color scheme: identical residues (black background), conserved substitutions (red background), and similar physicochemical properties (blue background). The aligned sequences correspond exactly to those used in the phylogenetic tree construction.

The effector SsEF83 paralogs within the *S. scitamineum* genome were identified through BLASTp searches. SsEF83 has two paralogs locating on the same contig, with sequence similarities of 0.4891 and 0.4363, respectively (Supplementary Table S2). Furthermore, orthologs of SsEF83 in Ustilaginales were identified. The closest ortholog of SsEF83 was identified in *S. reilianum*, showing a sequence identity of 0.3387 (Supplementary Table S3). However, no orthologs were identified in species belonging to Ascomycotina. The conserved motifs, phylogenetic tree and sequence alignment analysis of these orthologs were shown in Figure 2. The similar positions of their three conserved motifs further support sequence similarity.

**Figure 3:**
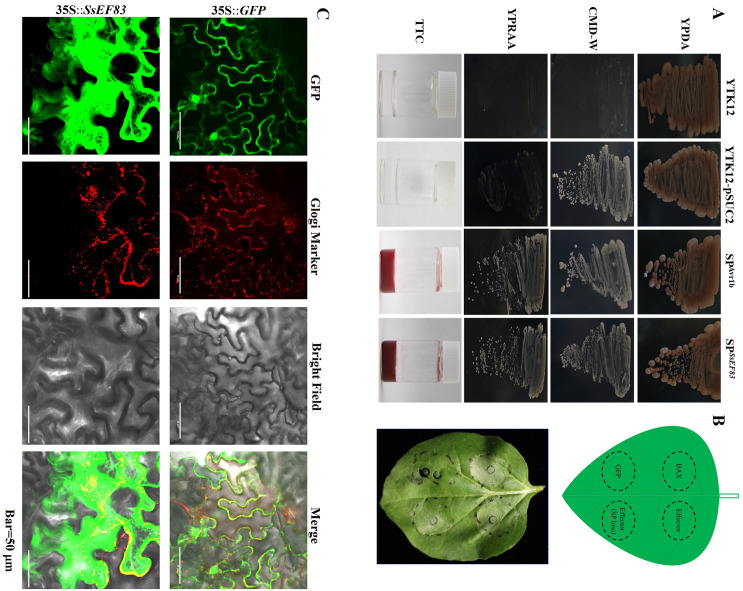
Functional analysis of the core effector *SsEF83* of *S. scitamineum*. **(A)** Validation of effector protein signaling peptide-mediated proteins secretion function. YPDA is yeast extract peptone dextrose agar; CMD-W is tryptophan-deficient medium; YPRAA is medium with raffinose as the sole carbon source; TTC, Triphenyl tetrazolium chloride; Positive control, SP^Avr1b^; Negative controls, YTK12 and YTK12-pSUC2. **(B)** SsEF83 induces PCD in *N. benthamiana* leaves. *Agrobacterium tumefaciens* containing 35S::effector or 35S::effector (SP loss) constructs was used to infiltrate *N. benthamiana* leaves. *A. tumefaciens* harboring 35S::GFP and *A. tumefaciens* harboring 35S::BAX were used as negative and positive controls, respectively. **(C)** Subcellular localization of the effector SsEF83. The subcellular localization assays were conducted in *N. benthamiana* leaves, and fluorescence was observed after 2 d of infiltration using laser confocal microscopy with a Glogi marker.

To investigate whether SsEF83 functions as a secreted protein, the signal peptide of SsEF83, as predicted by SignalP, was validated using the yeast secretion system. In this assay, the avirulence protein Avr1b from soybean *Phytophthora sojae*, known for its secretion capability, was employed as a positive control, while the pSUC2 empty vector was utilized as a negative control. As shown in Figure 3A, the yeast strain harboring the fusion gene with the SsEF83 signal peptide successfully grew on CMD-W medium, suggesting the successful transformation of the vector into the YTK12 strain. Furthermore, this yeast strain SP^SsEF83^ exhibited traits similar to the positive control, growing on YPRAA medium and changing the TTC solution. Therefore, it can be concluded that the SsEF83 signal peptide has secretion capability. Using a transient expression system in *N. benthamiana*, we performed the programmed cell death (PCD) assay, which revealed that SsEF83 induces PCD in *N. benthamiana* leaves independently of its signal peptide (Figure 3B). Additionally, subcellular localization analysis showed that the effector SsEF83 protein can be localized in the plant cytoplasm (Figure 3C).

### *SsEF83* is not required for fungal morphology, growth, and mating/filamentous in *S. scitamineum*

**Figure 4:**
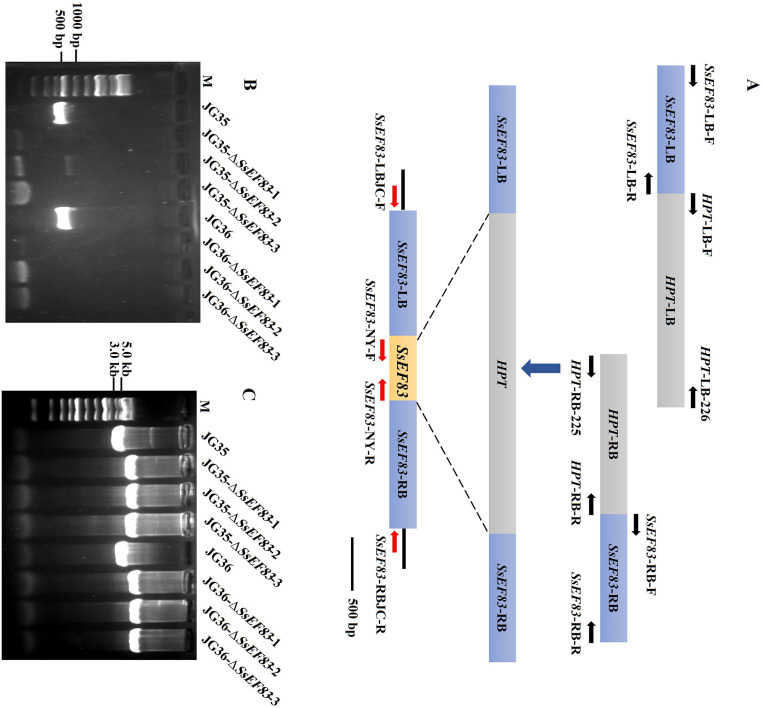
Construction and verification of *SsEF83* deletion mutants. **(A)** Schematic representation of the *SeEF83* gene deletion method. **(B)** PCR verification of deletion fragments. The primer pair SsEF83-NY-F/SsEF83-NY-R was used to amplify the SsEF83 coding region. **(C)** PCR verification of insertion fragments. The primer pair SsEF83-LBJC-F/SsEF83-RBJC-R were used to amplify the left and right ends of the insertion fragments.

To investigate the biological role of SsEF83 in *S. scitamineum*, the *SsEF83* deletion mutant was generated by replacing the *SsEF83* gene with a hygromycin-resistant gene. Three phenotypically identical knockout transformants of *SsEF83* in both mating types of *S. scitamineum* (designated as JG35-Δ*SsEF83*-1, JG35-Δ*SsEF83*-2, and JG35-Δ*SsEF83*-3; JG36-Δ*SsEF83*-1, JG36-Δ*SsEF83*-2, and JG36-Δ*SsEF83*-3) were successfully obtained and validated, as depicted in Figure 4. Subsequently, one of these knockout transformants was selected for complementation, resulting in the generation of the complemented strain (C-JG35-Δ*SsEF83* and C-JG36-Δ*SsEF83*; Supplementary Figure S2).

**Figure 5:**
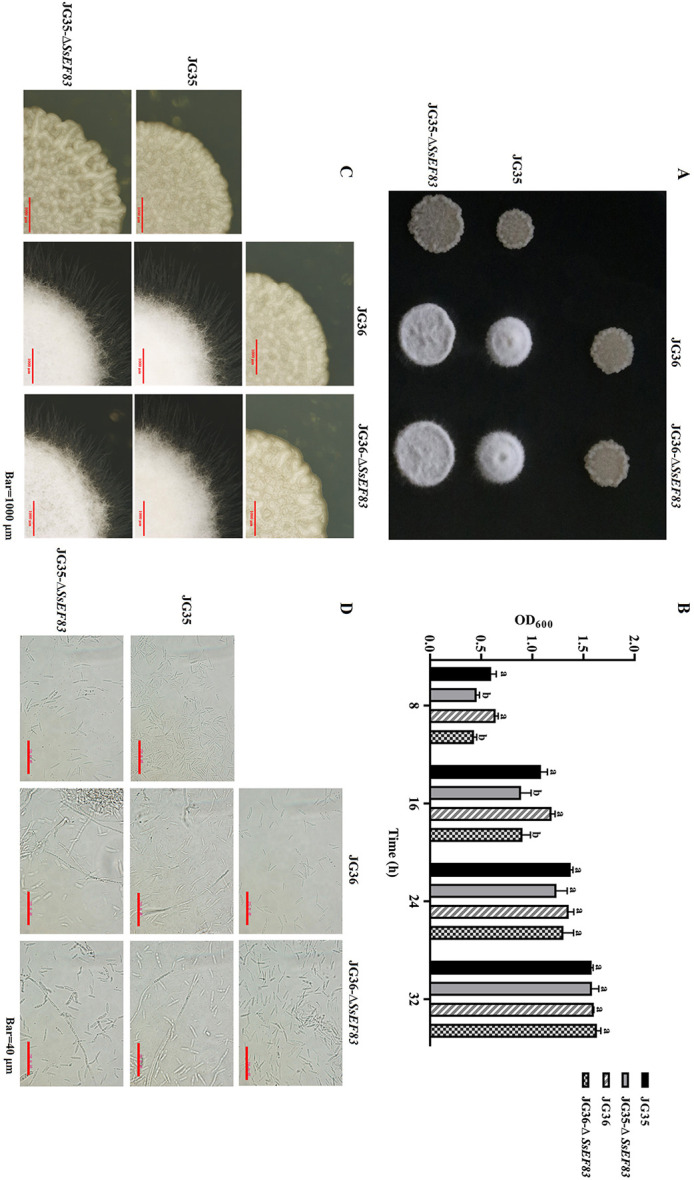
The deletion of *SsEF83* does not affect morphology, growth and mating/filamentous in *S. scitamineum*. **(A)** Wild-type and *SsEF83* deletion mutant strains were co-spotted on YEPS plates and incubated at 28°C for 72 h. The formation of white, fluffy colonies, dikaryotic hyphae and filamentous growth, confirms successful mating. **(B)** Growth rates of the wild-type, knockout mutant, and complemented mutant strains, represented as mean ± SE from three independent biological replicates. Values followed by the same letter are not significantly different as per Duncan's test (*p* < 0.05). **(C)** Enlarged view of the mating colony edge is shown in **(A)**. **(D)** Microscopic observation of the mating colony shown in **(A)**.

After 3 days of cultivation on YEPS plates, the morphology of the mutants was observed and photographed. As shown in Figure 5A, no significant deviations in colony morphology were observed in the mutant Δ*SsEF83* strain when compared to the wild-type JG35 and JG36. In liquid YEPS medium, the growth rate of the Δ*SsEF83* knockout strain was lower than that of the wild-type strain at 16 h post-inoculation. Meanwhile, their growth rates remained consistent at 32 h post-inoculation (Figure 5B), suggesting that *SsEF83* is not essential for the growth of *S. scitamineum*. Furthermore, when wild-type strains JG35 and JG36 were co-spotted on YEPS plates, they mated and formed dikaryotic filamentous hyphae, giving the colony a fluffy appearance. Similarly, colonies of JG35 × JG36-Δ*SsEF83*, JG35-Δ*SsEF83*×JG36, and JG35-Δ*SsEF83*×JG36-Δ*SsEF83* exhibited a phenotype indistinguishable from the wild type, characterized by the formation of white, fluffy mycelia (Figures 5A, C, D). Moreover, qRT-PCR analysis of genes, including those regulated by the cAMP-PKA and MAPK pathways, as well as genes at the *a* and *b* mating loci, revealed no significant expression differences between knockout and wild-type strains (Supplementary Figure S3). These findings indicate that the deletion of *SsEF83* does not influence mating/filamentous in *S. scitamineum*.

### *SsEF83* deletion mutants display attenuated virulence

**Figure 6:**
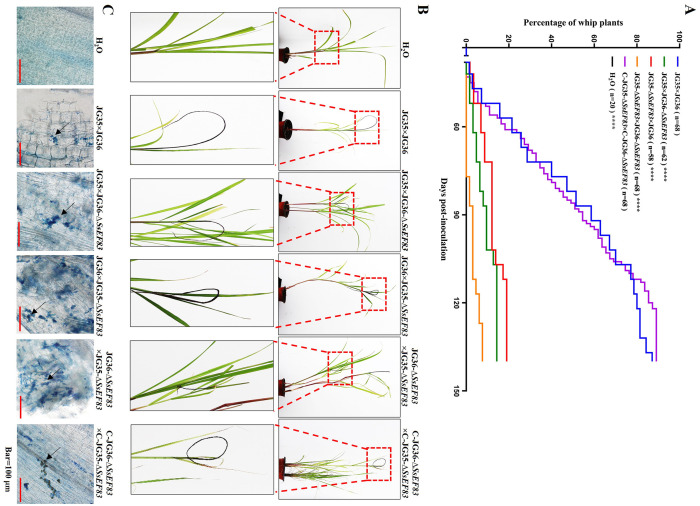
*SsEF83* deletion mutants exhibit reduced virulence. **(A)** The progression of whip development in sugarcane ROC22. Tissue culture-derived ROC22 were inoculated combinations of wild-type strains, *SsEF83* deletion mutants, and *SsEF83* complemented mutants by root dipping method. “*n*” indicates the total number of inoculated plants. The asterisk indicates a statistically significant difference from WT (****p < 0.0001; ANOVA with Tukey's multiple comparisons test). **(B)** Symptoms of sugarcane plantlets inoculated with *S. scitamineum*. Red dash-line boxed region was enlarged for a better view of black whip formation. **(C)** Histopathological analysis was conducted on the plantlets through dissection and subsequent staining with trypan blue. Arrows show the presence of fungal hyphae *in vivo*. Scale bars = 100 μm.

To evaluate the role of *SsEF83* in fungal virulence, we conducted inoculations on tissue culture seedlings of sugarcane variety ROC22 using wild-type strain (JG35 × JG36), water control (H_2_O), knockout mutants (JG35 × JG36-Δ*SsEF83*, JG35-Δ*SsEF83*×JG36, JG35-Δ*SsEF83*×JG36-Δ*SsEF83*), and complemented strains (C-JG35-Δ*SsEF83* × C-JG36-Δ*SsEF83*). As shown in Figure 6, the wild-type strains JG35 × JG36 inoculation resulted in 86.8% of the plantlets producing whips within 137 days. In comparison, the incidences of whip development for JG35-Δ*SsEF83*×JG36 and JG35 × JG36-Δ*SsEF83* were 18.9% (11/58) and 12.9% (8/62), respectively. By deleting one allele in the inoculation pair, the whip-producing percentage was reduced by 78–85%. Additionally, JG35-Δ*SsEF83* × JG36-Δ*SsEF83* showed a 10.3% incidence of whip development. The deletion of both alleles in the inoculation pair led to an 88% reduction in the whip generation. Furthermore, the complemented strain C-JG35-Δ*SsEF83*×C-JG36-Δ*SsEF83* restored the mutant virulence to a level comparable to that of the wild-type strain (Figures 6A, B). The presence of fungal hyphae was detected in all sugarcane plants with black whip symptoms through histopathological examination (Figure 6C). These findings collectively indicated that SsEF83 plays a critical role in the virulence of *S. scitamineum*.

### Identification of the host proteins interacting with *SsEF83*

**Figure 7:**
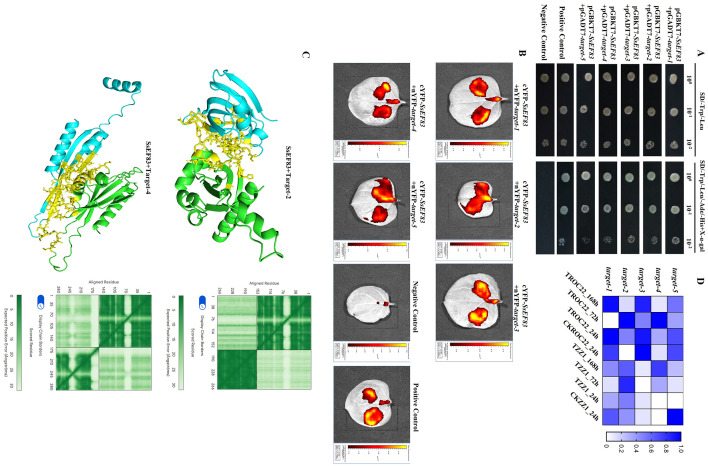
Identification of the host proteins interacting with SsEF83. **(A)** Y_2_H yeast cells containing pGADT7-host target and pGBKT7-SsEF83 could grow on both SD/–Trp/–Leu and SD/–Ade/–His/–Trp/–Leu+X-α-gal medium at three dilutions (10^0^, 10^−1^, and 10^−2^). Detailed information on interaction IDs is shown in Table 1. The yeast cells containing BD-p53 and AD-T7 were considered positive controls. The yeast cells containing BD-SsEF83 and AD were considered negative controls. **(B)** Validation of representative interactions between SsEF83 and host targets by bimolecular fluorescence complementarity in *N. benthamiana*. Observation of bimolecular fluorescence complementarity using bioluminescence imaging (IVIS Lumina LT, PerkinElmer). Each treatment was performed on three *N. benthamiana* leaves with two spots. **(C)** Analysis of the predicted interaction between SsEF83 and the target-2 and target-4 using AlphaFold 3. The left panel displays the interaction interface between SsEF83 and target, where the green region represents the SsEF83 protein, the blue region represents the target protein, and the yellow region indicates the interaction interface. The right panel shows the Predicted Position Error heatmap of the AlphaFold 3 prediction results. **(D)** Dual RNA-seq based expression profiles of the host targets in the smut-resistant variety ZZ1 and smut-susceptible variety ROC22 after *S. scitamineum* inoculation. Buds inoculated with sterile water after 24 h (CKZZ1_24h and CKROC22_24h) were used as controls and three replications of the experiments were performed. The heatmap was generated using GraphPad Prism v8.0.1.

To further investigate the function of the effector protein SsEF83 in the host plant, the yeast two-hybrid assay was applied to identify sugarcane proteins interacting with SsEF83. A cDNA library constructed from sugarcane ROC22 meristem infected with *S. scitamineum* was used for yeast two-hybrid screening. SsEF83 (without its signal peptide) was non-toxic to yeast cells and did not exhibit autoactivation activity (Supplementary Figure S4). From the library, five proteins were identified as interacting with SsEF83 (Figure 7A). Table 1 shows the information of five interacting proteins, including target-1 (protein NBR1 homolog), target-2 (peptidyl-prolyl cis-trans isomerase), target-3 (CBL-interacting protein kinase 32), target-4 (40S ribosomal protein S20-2), and target-5 (chaperone protein dnaJ GFA2). Furthermore, bimolecular fluorescence complementation assays in *N. benthamiana* confirmed interactions between SsEF83 and five host target proteins, respectively (Figure 7B). Additionally, the protein-protein interaction prediction results derived from AlphaFold 3 indicated substantial binding interactions between the SsEF83 protein and the target proteins. Notably, the predicted template modeling scores (pTM) for both target-2 and target-4 surpassed 0.5. Specifically, the interaction interface between SsEF83 and target-2 is characterized by the presence of 38 hydrogen bonds and 49 hydrophobic interactions. In contrast, the interaction interface between SsEF83 and target-4 forms a more complex network, comprising 68 hydrogen bonds and 85 hydrophobic interactions (Figure 7C). The findings suggest that SsEF83 establishes stable interaction interfaces with the two target proteins, thereby offering a structural basis for further investigation into its molecular mechanisms. Furthermore, co-expression of SsEF83 and target2/4 showed that the two targets failed to suppress SsEF83-induced PCD in *N. benthamiana* leaves (Supplementary Figure S5).

**Table 1:** Host targets of the effector SsEF83.

**Protein designation**	**Reference protein**	**Annotation**	**Identity (%)**
Target-1	XP_002454107.1	Protein NBR1 homolog [Sorghum bicolor]	91.64
Target-2	QIJ58750.1	Peptidyl-prolyl cis-trans isomerase [Saccharum hybrid cultivar FN02-3924]	95.96
Target-3	NP_001345807.1	CBL-interacting protein kinase 32 [Zea mays]	98.42
Target-4	XP_014661210.1	40S ribosomal protein S20-2 [Setaria italica]	99.13
Target-5	XP_002437726.1	Chaperone protein dnaJ GFA2, mitochondrial [Sorghum bicolor]	96.27

Based on our previous dual RNA-seq data of sugarcane infected by *S. scitamineum* (Liu et al., 2022), differential expression patterns of these target proteins were shown. In the susceptible variety ROC22, the expression levels of all five targets were elevated 24 h post-inoculation relative to those observed in the resistant variety ZZ1. Moreover, the expression levels of the target-2 and target-4 genes were significantly increased 72 h post-infection of ROC22 by smut, in comparison to the control group. Likewise, elevated expression levels of these two genes were observed in the transcriptome analysis of ZZ1 (Figure 7D). These changes in expression levels indicate a potential association between the two target proteins and the susceptibility or resistance of sugarcane to smut.

## Discussion

Obligate fungal pathogens, such as *S. scitamineum*, secrete effectors and cell wall-degrading enzymes to facilitate host invasion and colonization (Valent and Khang, 2010; Guzmán-Guzmán et al., 2017). Certain effectors are transcriptionally induced during infection through the activation of specific transcription factors (Kleemann et al., 2012; Lanver et al., 2014). Effector proteins are critical virulence factors in fungi, promoting the infection and colonization of pathogenic fungi by modulating host immune responses, suppressing defense signaling pathways, or disrupting cellular functions (Oliveira-Garcia et al., 2024). The identification of novel effectors in fungal pathogens is challenging due to their low degree of conservation (Stergiopoulos and De Wit, 2009; Lo Presti et al., 2015). However, identifying fungal effectors is becoming easier with the development of omics studies. The Pele1 effector in *S. scitamineum* was identified using dual RNA-seq combined with weighted gene co-expression network analysis, suggesting that this method can be used to identify important effectors (Ling et al., 2022; Dai et al., 2024). identified 169 effector candidates containing conserved domains (e.g., LysM, CFEM, Chitin_bind) in the rice endophytic fungus *Falciphora oryzae* through bioinformatics prediction and functional validation. Some of these effectors were found to activate or inhibit cell death in tobacco, and their gene expression was significantly upregulated during the infection process. Through transcriptomic and proteomic analyses, (Kahar et al. 2024) predicted 210 candidate effector proteins (CEPs) from the apple canker pathogen *Valsa mali* and identified 146 CEP-encoding genes that were differentially expressed during the infection process. Through comparative genomics, (Khodadadi et al. 2024) identified 390 to 581 predicted effectors in the genomes of four *Colletotrichum* spp. (*C. chrysophilum, C. noveboracense, C. nupharicola*, and *C. fioriniae*).

After infecting sugarcane, *S. scitamineum* secretes effector proteins and other virulence factors, engaging in molecular-level interactions with the host. Currently, research on the effector proteins of *S. scitamineum* remains limited. In *S. scitamineum*, previous studies have reported upregulation of effector-encoding genes (*g3890, g5159*, and *SsPele1*) during host infection (Teixeira-Silva et al., 2020; Ling et al., 2022). Through proteomic analysis, Barnabas et al. discovered that infection of a susceptible sugarcane cultivar by *S. scitamineum* led to the involvement of 53 differentially abundant proteins in defense, metabolism, and cell division processes in the shoot apical meristem. They identified the pathogen effector chorismate mutase and noted a significant rise in phenylalanine ammonia lyase activity and transcript levels. Additionally, the abundance of seven candidate proteins correlated with their corresponding transcript expression levels (Barnabas et al., 2016). Overall, research on effector proteins of *S. scitamineum* is still in its early stages, with limited breadth and depth of investigation. Based on our dual RNA-seq data from *S. scitamineum* and sugarcane interactions, we identified the effector protein SsEF83. This protein exhibits classic effector characteristics: it induces PCD in *N. benthamiana*, contains a signal peptide facilitating secretion, and is upregulated during plant infection. The functional annotation of protein SsEF83 is an unknown protein, and its homologous proteins have not been reported.

Gene knockout technology enables in-depth investigation into the functional roles of effector proteins, clarifying their correlation with pathogen virulence. (Seitner et al. 2018) successfully identified *Cce1*, a critical effector gene in *M. maydis*, as essential for pathogenicity. Knockout mutants of *Cce1* were blocked during early maize infection, accompanied by significantly elevated callose deposition in host tissues. (Chen et al. 2025) demonstrated that the *R. solanacearum* effector RipAU enhances pathogenicity by targeting the peanut subtilisin-like protease AhSBT1.7, which modulates the interaction between AhSBT1.7 and pectin methylesterase AhPME4 to promote PME-mediated cell wall degradation. Notably, the Δ*RipAU* mutant completely lost pathogenicity. (Meng et al. 2024) revealed that the *Valsa mali* effector VmSpm1 degrades the host protein MdPYL4, thereby suppressing jasmonic acid (JA) signaling and weakening apple resistance to canker, whereas MdPYL4 overexpression enhances disease resistance by promoting JA biosynthesis. Our knockout study of *SsEF83* demonstrated that this gene does not affect the growth, mating ability, or filamentous development of *S. scitamineum*. However, deletion of *SsEF83* significantly reduced the fungal virulence: single-knockout mutants exhibited 78% to 85% virulence loss, while double-allele knockout mutants showed 88% virulence attenuation. The complementation of *SsEF83* restored wild-type virulence, confirming its role as a critical virulence factor in *S. scitamineum*. Besides, we identified two paralogs of *SsEF83* in the *S. scitamineum* genome. Further research is required to investigate whether the two paralogs affect the growth and virulence of *S. scitamineum*.

Among the five interacting sugarcane proteins identified in this study, the NBR1 protein has been implicated in the regulation of PCD (Timotijević et al., 2010). Additionally, peptidyl-prolyl cis-trans isomerase has been shown to enhance resistance in cotton against *Verticillium dahliae*, effectively inhibiting fungal spore germination and hyphal growth (Yang et al., 2019). Moreover, CBL-interacting protein kinase 32 (CIPK32) plays a role in stabilizing intracellular reactive oxygen species (ROS) by modulating ROS production and scavenging, thereby strengthening pathogen resistance in plants (Ma et al., 2020). In rice, the chaperone protein DnaJ exerts a negative effect on immunity by enhancing the transcriptional activity of the NAC transcription factor MNAC3. MNAC3 binds to CACG cis-elements to activate the transcription of immune-suppressive target genes *OsINO80, OsJAZ10*, and *OsJAZ11*, thereby dampening the plant immune response (Wang et al., 2024). As SsEF83 localizes within the host cytoplasm, it likely interacts with these resistance-related proteins to suppress immune responses, potentially enhancing *S. scitamineum* virulence. Given the extended timeframe of *S. scitamineum* infection—ranging from weeks to months—further research is necessary to assess the role of *SsEF83* and other effectors during mid- and late-infection stages to gain a comprehensive understanding of the pathogen's virulence strategies. These findings suggest that SsEF83 may interact with these host proteins to alter immune-related pathways, shedding light on possible mechanisms by which *S. scitamineum* may manipulate host defenses.

In conclusion, we identified a critical secreted effector, SsEF83, which localizes in the plant cytoplasm and induces PCD in *N. benthamiana* leaves. The expression of *SsEF83* was significantly upregulated during *S. scitamineum* infection of sugarcane, and its deletion markedly attenuated fungal virulence. Furthermore, we identified five sugarcane proteins interacting with SsEF83, which are associated with processes such as PCD, ROS generation, signaling transduction, and transcriptional regulation. This study provides valuable insights into the interactions between sugarcane and its smut fungus, advancing our understanding of effector-mediated pathogenicity in *S. scitamineum*.

## Data Availability

The original contributions presented in the study are included in the article/Supplementary material, further inquiries can be directed to the corresponding author.
