# Supplementary material for: Decoding sugarcane smut: the role of effector SsEF83 in fungal virulence and plant interaction
Source: Front Microbiol. 2025 Aug 18;16:1586720. doi: 10.3389/fmicb.2025.1586720 (PMC12399601; doi:10.3389/fmicb.2025.1586720)
Supplement: Supplementary file 2 [file Presentation_1.pdf]

Fig. S1. Predicting the signal peptide of the SsEF83 protein. SignalP predicts it to be a secreted protein, with the putative N-terminal secretion signal expected to be cleaved after 29 amino acids.

Fig. S2. Construction and verification of SsEF83 complementation mutants. (A) PCR verification of complementation fragments. The primer pair SsEF83-NY-F/SsEF83-NY-R was used to amplify the SsEF83 coding region. (B) Semi-quantitative PCR verification of complementation fragments. The primer pair SsEF83-NY-F/SsEF83-NY-R was used to amplify the SsEF83 coding region. (C) Validation of the internal reference in semi-quantitative PCR in (B). PCR amplification was performed using the primer pair S10-F/S10-R.

Fig. S3. The deletion of *SsEF83* does not affect the expression of genes essential for mating and filamentous growth in the dikaryotic stage. qRT-PCR was employed to quantify the accumulation of target genes, using the actin gene of *S. scitamineum* as an endogenous control. Data are presented as the mean ± SE from three independent biological replicates, with each replicate consisting of three technical replicates. The p-value was calculated using ANOVA with Tukey's multiple comparisons test for each gene between the wild-type and *SsEF83* mutant strains. There was no significant difference in the result.

Fig. S4. Toxicity test and Autoactivate test of baits. (A) Toxicity test of SsEF83 bait protein. The colonies were grown on SD/-Trp medium and incubated at 30 °C for 24 h. (B) The colonies were on SD/-Trp/-Leu medium and SD/-Trp/-Leu/-Ade/-His medium at 30 °C for 48 h.

Fig. S5. Co-expression of SsEF83 and target2/4 showed that the two targets failed to suppress SsEF83-induced PCD in *N. benthamiana* leaves. The *A. tumefaciens* containing 35S::SsEF83 and target-2 or target-4 protein was infiltrated into the leaves of *N. benthamiana*. The *A. tumefaciens* harboring 35S::BAX was used as a

positive control.
