# Supplementary figures and images for: Decoding sugarcane smut: the role of effector SsEF83 in fungal virulence and plant interaction
Source: Front Microbiol. 2025 Aug 18;16:1586720. doi: 10.3389/fmicb.2025.1586720 (PMC12399601; doi:10.3389/fmicb.2025.1586720)

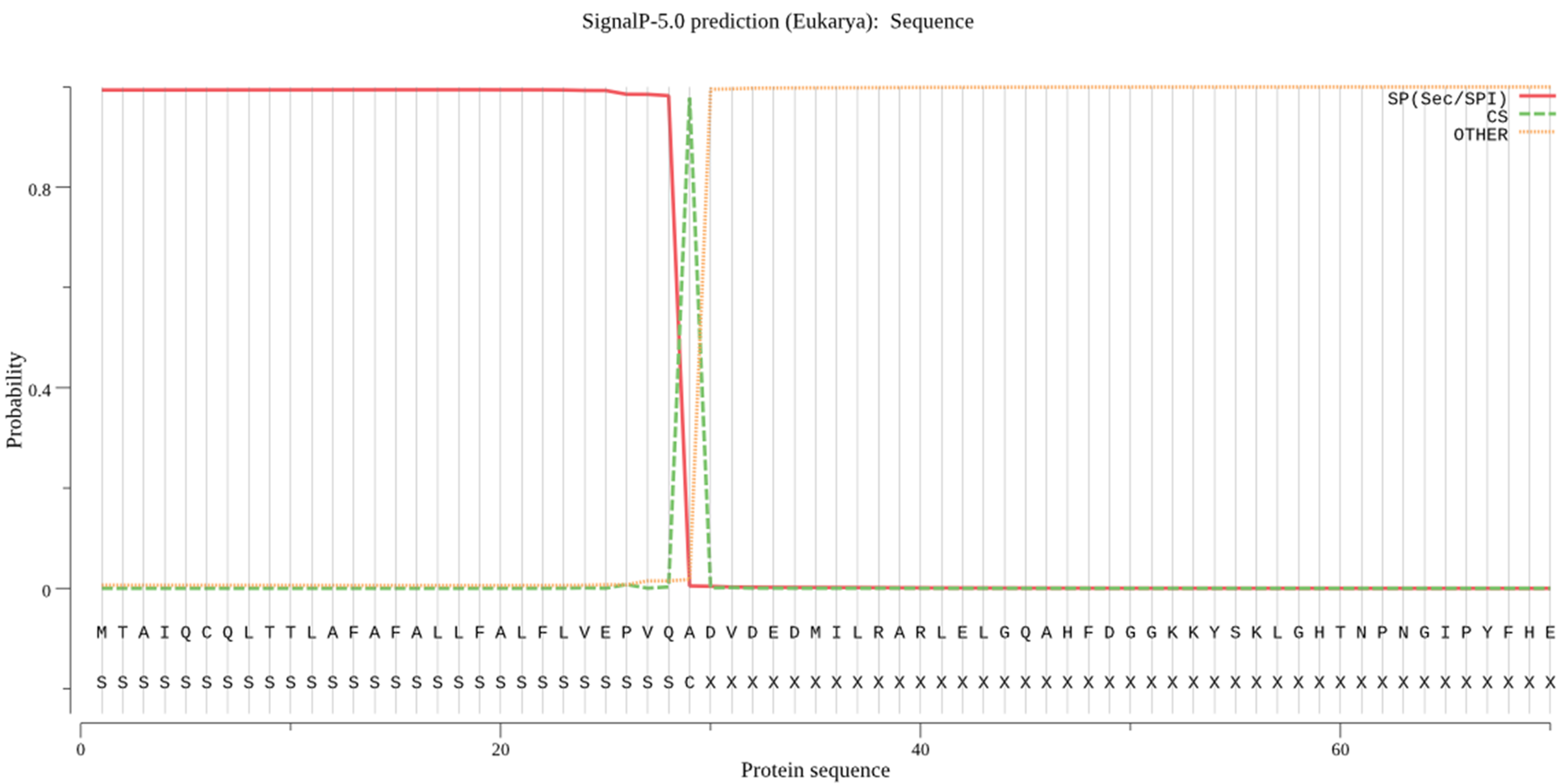

Supplement: Supplementary file 3 [file Image_1.png]

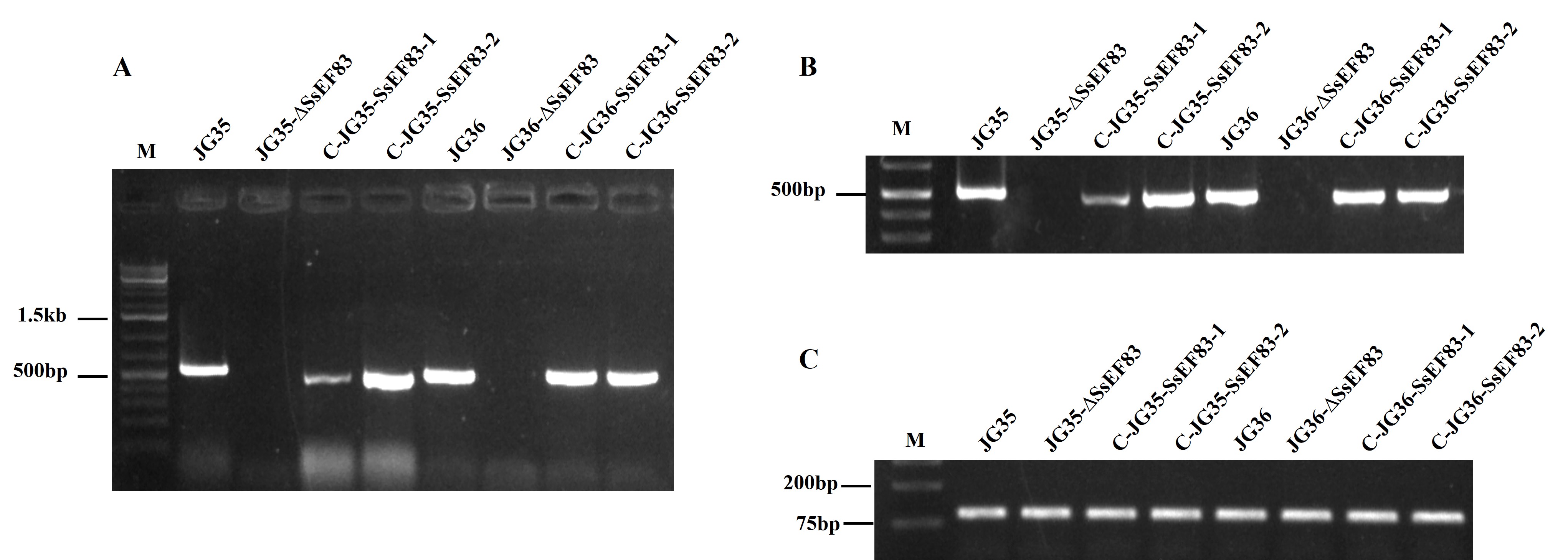

Supplement: Supplementary file 4 [file Image_2.jpg]

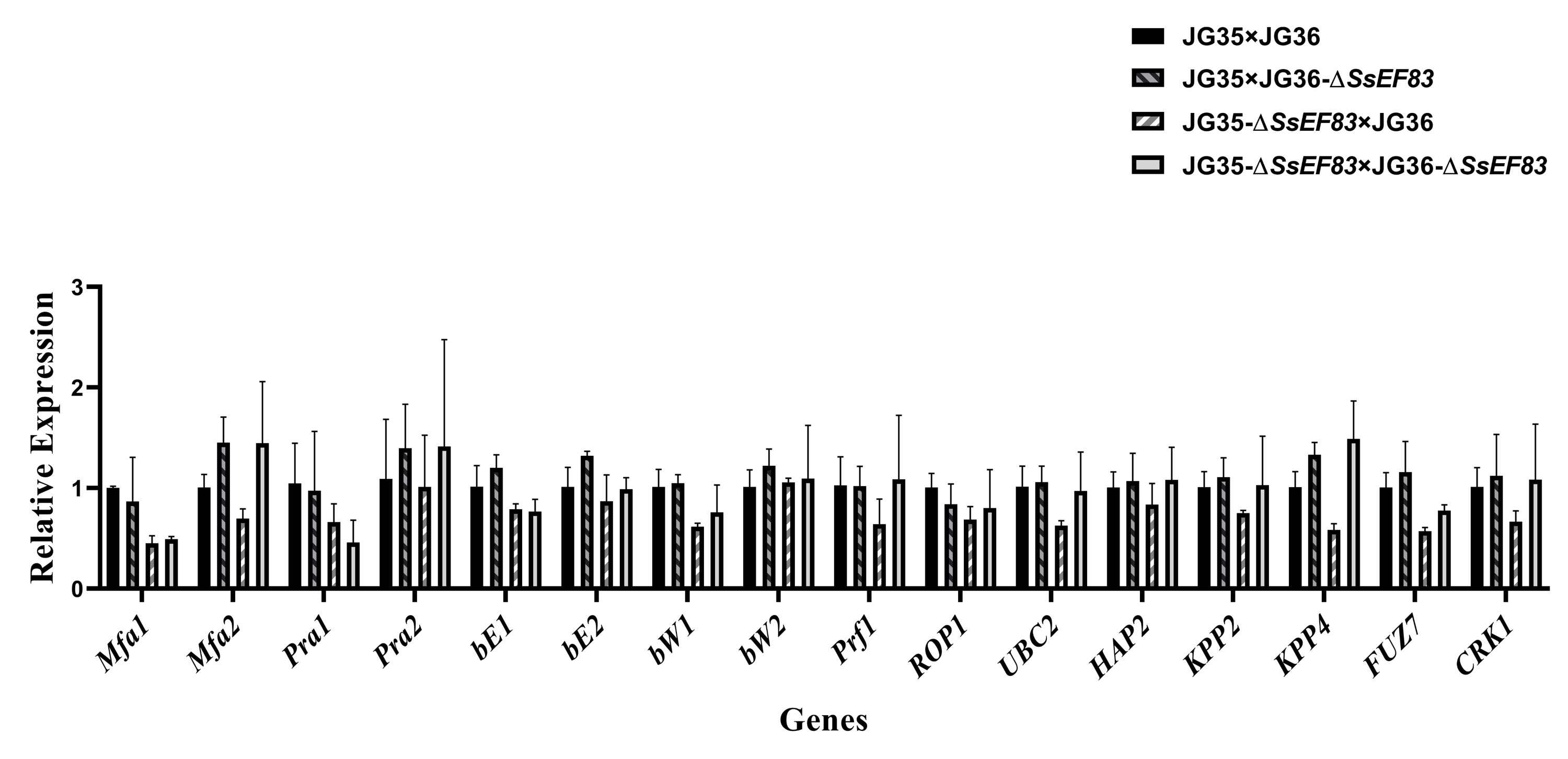

Supplement: Supplementary file 5 [file Image_3.jpg]

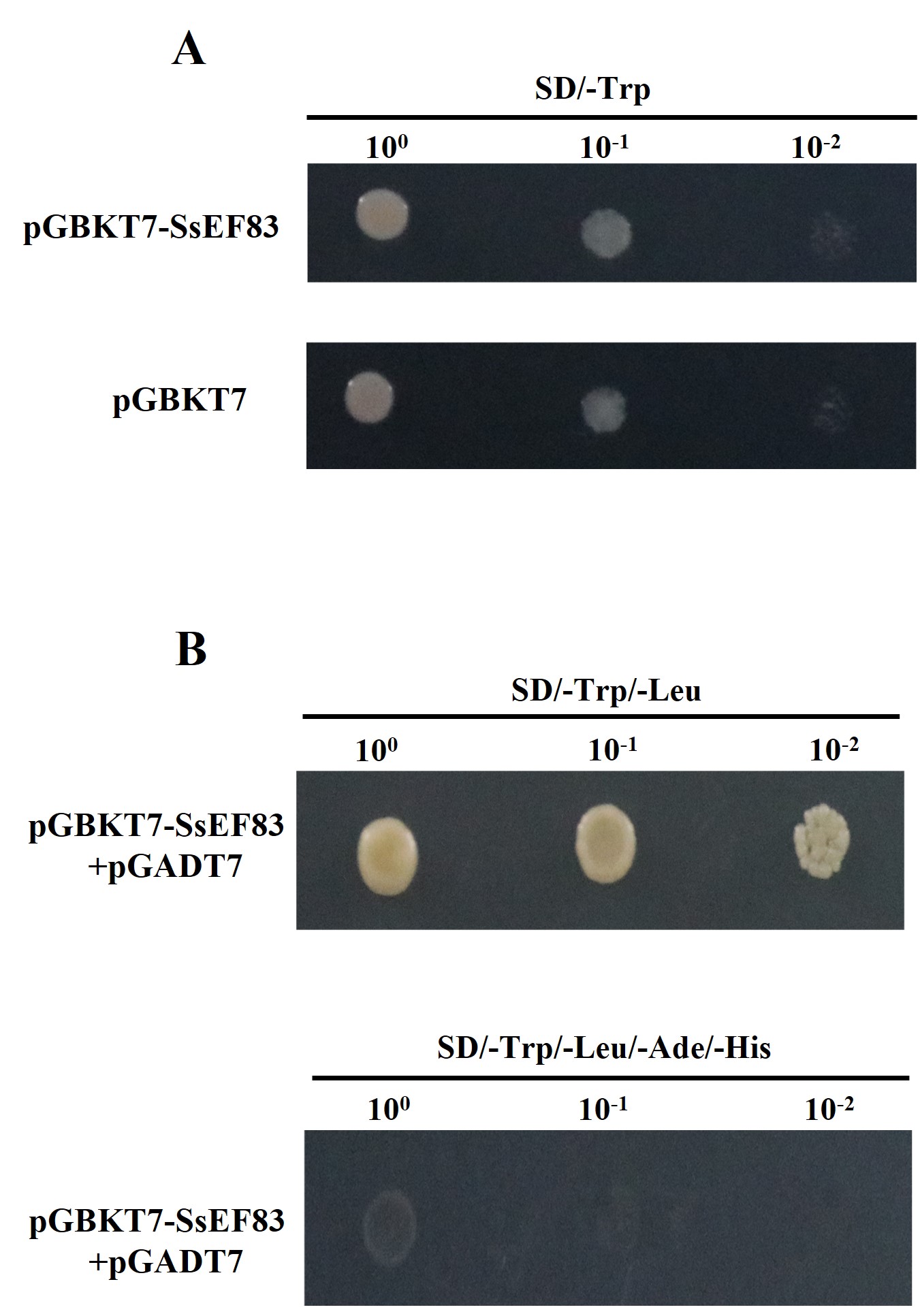

Supplement: Supplementary file 6 [file Image_4.jpg]

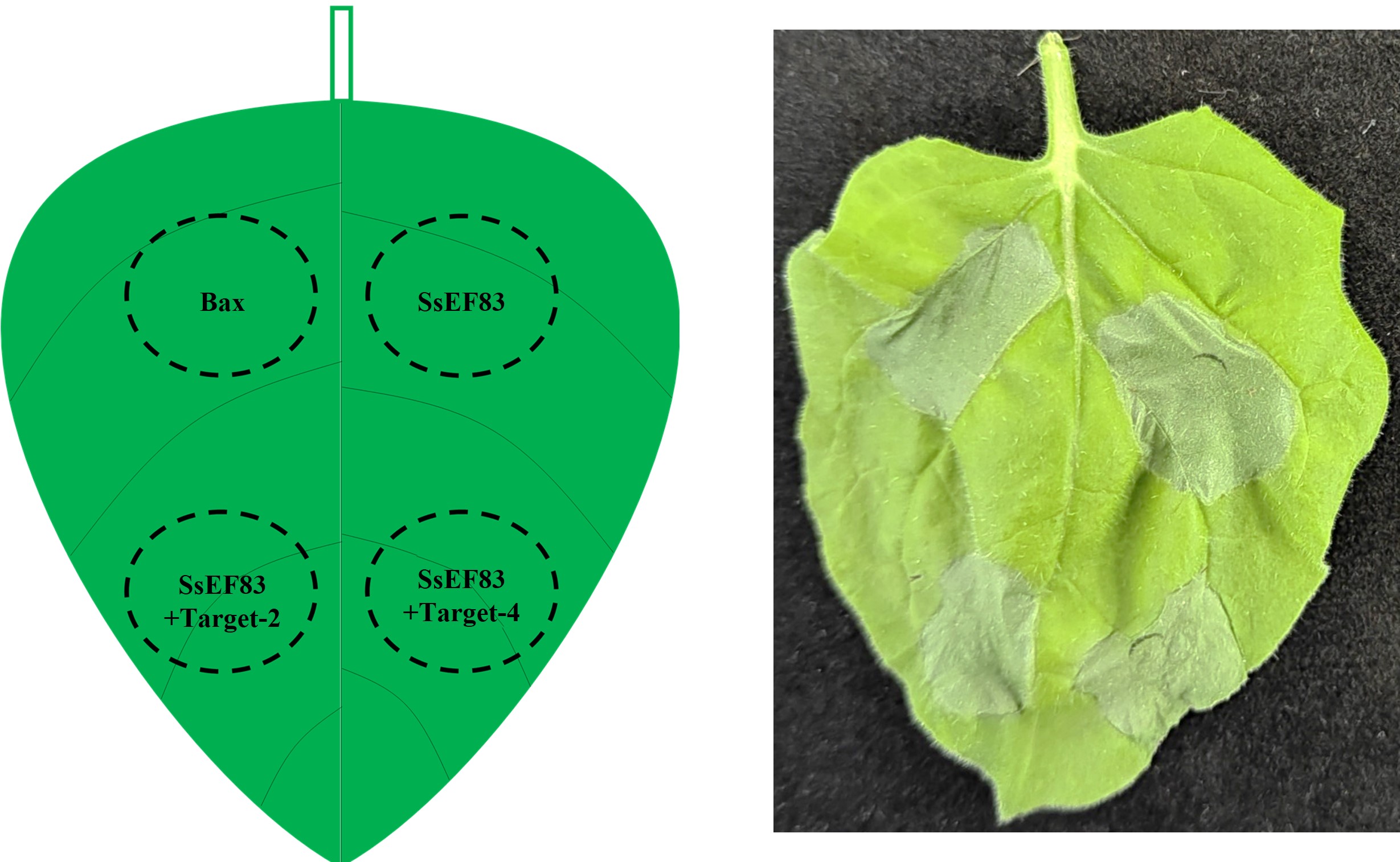

Supplement: Supplementary file 7 [file Image_5.jpg]
